# CSF 14-3-3β is associated with progressive cognitive decline in Alzheimer’s disease

**DOI:** 10.1093/braincomms/fcad312

**Published:** 2023-11-22

**Authors:** Qiang Qiang, Loren Skudder-Hill, Tomoko Toyota, Zhe Huang, Wenshi Wei, Hiroaki Adachi, Alzheimer’s Disease Neuroimaging Initiative

**Affiliations:** Department of Neurology, Cognitive Disorders Center, Huadong Hospital, Fudan University, 200040 Shanghai, China; Department of Neurology, University of Occupational and Environmental Health School of Medicine, 807-8555 Kitakyushu, Japan; Yuquan Hospital, Tsinghua University School of Clinical Medicine, 100084 Beijing, China; School of Medicine, University of Auckland, 1023 Auckland, New Zealand; Department of Neurology, University of Occupational and Environmental Health School of Medicine, 807-8555 Kitakyushu, Japan; Department of Neurology, University of Occupational and Environmental Health School of Medicine, 807-8555 Kitakyushu, Japan; Department of Neurology, Cognitive Disorders Center, Huadong Hospital, Fudan University, 200040 Shanghai, China; Department of Neurology, University of Occupational and Environmental Health School of Medicine, 807-8555 Kitakyushu, Japan

**Keywords:** Alzheimer’s disease, 14-3-3β, CSF, biomarker, dementia

## Abstract

Alzheimer’s disease is a neurodegenerative disorder characterized pathologically by amyloid-beta plaques, tau tangles and neuronal loss. In clinical practice, the 14-3-3 isoform beta (β) is a biomarker that aids in the diagnosis of sporadic Creutzfeldt–Jakob disease. Recently, a proteomics study found increased CSF 14-3-3β levels in Alzheimer’s disease patients, suggesting a potential link between CSF 14-3-3β and Alzheimer’s disease. Our present study aimed to further investigate the role of CSF 14-3-3β in Alzheimer’s disease by analysing the data of 719 participants with available CSF 14-3-3β measurements from the Alzheimer’s Disease Neuroimaging Initiative. Higher CSF 14-3-3β levels were observed in the mild cognitive impairment group compared to the cognitively normal group, with the highest CSF 14-3-3β levels in the Alzheimer’s disease dementia group. This study also found significant associations between CSF 14-3-3β levels and CSF biomarkers of p-tau, t-tau, pTau/Aβ42 ratios and GAP-43, as well as other Alzheimer’s disease biomarkers such as Aβ-PET. An early increase in CSF 14-3-3β levels was observed prior to Aβ-PET–positive status, and CSF 14-3-3β levels continued to rise after crossing the Aβ-PET positivity threshold before reaching a plateau. The diagnostic accuracy of CSF 14-3-3β (area under the receiver operating characteristic curve = 0.819) was moderate compared to other established Alzheimer’s disease biomarkers in distinguishing cognitively normal Aβ pathology–negative individuals from Alzheimer’s disease Aβ pathology–positive individuals. Higher baseline CSF 14-3-3β levels were associated with accelerated cognitive decline, reduced hippocampus volumes and declining fluorodeoxyglucose-PET values over a 4-year follow-up period. Patients with mild cognitive impairment and high CSF 14-3-3β levels at baseline had a significantly increased risk [hazard ratio = 2.894 (1.599–5.238), *P* < 0.001] of progression to Alzheimer’s disease dementia during follow-up. These findings indicate that CSF 14-3-3β may be a potential biomarker for Alzheimer’s disease and could provide a more comprehensive understanding of the underlying pathological changes of Alzheimer’s disease, as well as aid in the diagnosis and monitoring of disease progression.

## Teaser

Qiang *et al*. report that increased CSF 14-3-3β levels in patients with Alzheimer’s disease correlate with cognitive decline and biomarkers of the disease, suggesting CSF 14-3-3β as a potential biomarker and tool for monitoring disease progression.

## Graphical Abstract

**Graphical Abstract:**
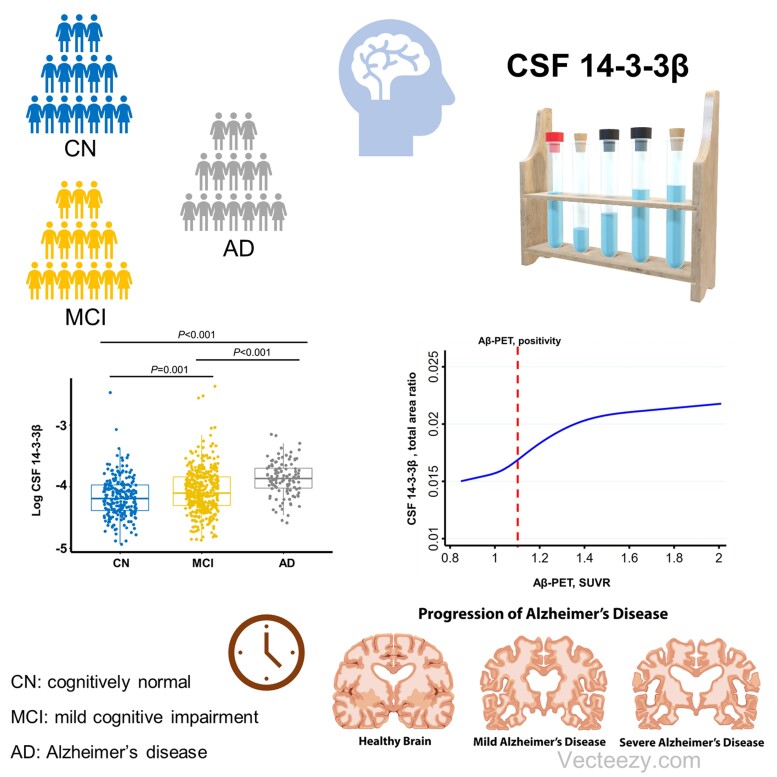

## Introduction

Alzheimer’s disease is a chronic neurodegenerative disorder that affects millions of people worldwide, especially those over the age of 65.^1^ Alzheimer’s disease is characterized pathologically by the accumulation of β-amyloid plaques and tau tangles in the brain, as well as neuronal loss and brain atrophy.^2^ Biomarkers are objective and measurable indicators that can reflect the pathological changes underlying disease, and the core biomarkers of Alzheimer’s disease can be categorized into three main types: biomarkers of β-amyloid deposition, biomarkers of tau pathology and biomarkers of neurodegeneration. These biomarkers can be detected in biological fluids, such as CSF, or through neuroimaging techniques, such as MRI or PET scans, which can be utilized to classify patients according to the amyloid/tau/neurodegeneration [AT(N)] biomarker system.^3^ While CSF and PET biomarkers for β-amyloid plaques and tau pathology have high accuracy in detecting Alzheimer’s disease pathology, their feasibility in clinical diagnostic practice is limited due to the high cost and low availability of required tools and equipment.^4^ One potential approach to overcome the above issues is to explore whether some established biomarkers, which are already used in routine clinical practice, could aid in the diagnosis and prediction of disease progression of Alzheimer’s disease patients.

The 14-3-3 proteins are a family of highly conserved regulatory proteins that play important roles in various cellular processes, including signal transduction, cell cycle control and apoptosis.^5^ The 14-3-3 proteins are abundantly expressed in brain tissue and constitute ∼1% of total soluble brain protein.^6^ There are seven isoforms of the 14-3-3 protein family, including beta (β), gamma (γ), epsilon (ε), zeta (ζ), eta (η), theta (θ) and sigma (σ). In humans, the 14-3-3 isoform β is encoded by the YWHAB gene, which is located on the 20th chromosome.^7^ Testing for 14-3-3β in CSF has already been established as an effective method aiding the diagnosis of prion diseases, such as Creutzfeldt–Jakob disease, and multiple studies have confirmed the utility of this biomarker clinically for the diagnosis of sporadic Creutzfeldt–Jakob disease.^8–12^ A recent study using integrative proteomics to investigate new CSF biomarkers in Alzheimer’s disease identified hundreds of proteins with significantly altered abundance in CSF. Among these proteins, CSF 14-3-3β levels were significantly increased in Alzheimer’s disease patients.^13^

In this study, we further explore the characteristics of CSF 14-3-3β in Alzheimer’s disease with participants from the Alzheimer’s Disease Neuroimaging Initiative (ADNI), and 719 participants with available CSF 14-3-3β measurements at baseline were involved in this study. We aimed to evaluate whether CSF 14-3-3β levels were elevated in Alzheimer’s disease, the associations of CSF 14-3-3β with other biomarkers of Alzheimer’s disease and the diagnostic accuracy of CSF 14-3-3β and its association with Alzheimer’s disease progression and cognitive decline.

## Materials and methods

### Participants

All participants in this study were sourced from the ADNI. Launched in 2003 as a public–private project under the leadership of principal investigator Michael W. Weiner, MD, the ADNI aims to facilitate research on Alzheimer’s disease by developing clinical, genetic, MRI/PET imaging and CSF/blood biomarkers for early Alzheimer’s disease diagnoses and monitoring of disease progression. The ADNI study as a multi-centred investigation was approved by ethics committees of all involved institutions and was conducted according to relevant guidelines and regulations. Authorized written informed consent was obtained from all study participants. The data analysed in this report were downloaded from the ADNI database (http://adni.loni.usc.edu/) in November 2022. This study included 719 participants with available CSF 14-3-3β measurements at baseline from the ADNI, ranging from clinically diagnosed cognitively normal (CN) to mild cognitive impairment (MCI) and Alzheimer’s disease dementia. For gender demographics, males constituted 43.61% of the CN group (99 of 227 participants), 54.88% of the MCI group (208 of 379 participants) and 60.18% of the Alzheimer’s disease dementia group (68 of 113 participants). The criteria for inclusion and exclusion as well as the specific ADNI diagnostic criteria for differentiating CN, MCI and Alzheimer’s disease participants have been previously outlined.^14^

### CSF 14-3-3β measurements

CSF 14-3-3β levels were analysed using targeted proteomics assay by mass spectrometry (MS) at the Department of Neurology, Emory University School of Medicine. Crude CSF samples were reduced and alkylated, then denatured. CSF proteins were digested by Lys-C and trypsin. After digestion, peptides were analysed by the Agilent 1290 Infinity II liquid chromatography system coupled with the TSQ Altis Triple Quadrupole Mass Spectrometer (Thermo Fisher Scientific), and the mass spectrometer was set to acquire data using the single reaction monitoring approach. Total area ratios were calculated for relative quantification of CSF 14-3-3β peptides 44-51 (NLLSVAYK) and 63-70 (VISSIEQK). Detailed methods of the CSF 14-3-3β quantification can be downloaded from the ADNI database (http://adni.loni.usc.edu/).

### CSF and plasma biomarker measurements

The levels of Aβ42, total tau (t-tau) and phosphorylated tau 181 (p-tau) in the CSF samples were analysed at the ADNI Biomarker Core laboratory at the University of Pennsylvania Medical Center. CSF Aβ42, CSF t-tau and CSF p-tau were quantified using electrochemiluminescence immunoassays on a fully automated Elecsys cobas e 601 instrument as previously described.^15,16^ The positivity of Aβ status and tau status were defined using the published cut-off values,^17^ which specified that participants with a baseline CSF p-tau/Aβ42 ratio >0.028 were considered to have a positive Aβ status, and participants with baseline CSF p-tau levels >27 pg/mL were assigned a positive tau status. The levels of CSF growth-associated protein 43 (GAP-43) were measured at the Clinical Neurochemistry Lab, University of Gothenburg, Sweden, through an in-house enzyme-linked immunosorbent assay (ELISA) method as previously described.^18^

Measurements of plasma Aβ42 and Aβ40 levels were quantified at Bateman lab, Washington University School of Medicine. An anti-Aβ mid-domain antibody (HJ5.1) was used to isolate the specific Aβ isoforms in the KingFisher (Thermo) automated immunoprecipitation platform. The sample was then digested with Lys-N protease and analysed through liquid chromatography tandem MS, as previously described.^19^ The Clinical Neurochemistry Lab at the University of Gothenburg, Sweden, analysed both plasma p-tau 181 and neurofilament light (NfL) levels using the single molecule array (Simoa) technique.^20,21^ Plasma p-tau181 levels were measured by combining two monoclonal antibodies (Tau12 and AT270) targeting N-terminal to mid-domain forms of p-tau 181. Plasma NfL levels were measured using a combination of monoclonal antibodies.

### Neuroimaging

This study utilized 3.0 T MRI scanners for brain imaging. FreeSurfer version 5.1 was used to quantify regional volumes based on the 2010 Desikan–Killany atlas.^22^ The input for FreeSurfer was T_1_-weighted MRI images (MPR or IR-SPGR) in the NiFTI format. During analyses, we used data of hippocampus volumes and adjusted for intracranial volume.

Aβ burden was estimated by amyloid PET using florbetapir (AV45) tracer, and amyloid PET image data were processed, and summary data were updated regularly by the Helen Wills Neuroscience Institute, University of California Berkeley, and Lawrence Berkeley National Laboratory. The image data were processed with FreeSurfer to define a cortical summary that included frontal, anterior/posterior cingulate, lateral parietal and lateral temporal regions. For cross-sectional analyses, the whole cerebellum was defined as the reference region. Summary global florbetapir standard uptake value ratios (SUVRs) were calculated, and amyloid PET results were defined as positive if global SUVRs were higher than 1.11.^23,24^ Fluorodeoxyglucose (FDG)-PET image data were processed by the Helen Wills Neuroscience Institute, University of California Berkeley, and Lawrence Berkeley National Laboratory. A set of pre-defined regions of interest (MetaROIs) were developed based on literature review that was indicative of MCI and Alzheimer’s disease pathological metabolic changes. These regions included the left angular gyrus, right angular gyrus, bilateral posterior cingulate gyrus, left inferior temporal gyrus and right inferior temporal gyrus and were normalized by the pons and cerebellar vermis reference regions.^25^

### Cognition measurement

The participants’ general cognition level in this cohort was assessed using the Mini-Mental State Examination (MMSE) and the Clinical Dementia Rating Scale-Sum of Boxes (CDR-SB).

### Statistical analysis

Differences in baseline characteristics between the Aβ-negative and Aβ-positive groups were assessed using the Wilcoxon rank-sum tests for continuous variables and Pearson’s *χ*^2^ tests for categorical variables. The CSF 14-3-3β data were found to have a skewed distribution based on visual inspection of the histogram and were log transformed to obtain a data set with a normal distribution. Multiple group comparisons of log-transformed CSF 14-3-3β data were conducted using one-way ANOVA and the Tukey *post hoc* test. Pearson correlations were calculated between baseline log-transformed CSF p-tau and log-transformed CSF 14-3-3β. Linear regression models were used to test associations between fluid biomarkers with CSF 14-3-3β, and analyses were adjusted for age, sex, years of education and APOE ε4 status. We applied a cubic spline model to investigate the trajectories of CSF 14-3-3β as a function of Aβ-PET and used Aβ-PET as a proxy of disease progression along the Alzheimer’s disease continuum. The cubic spline model was estimated by using the postrcspline command in Stata software. Receiver operating characteristic (ROC) analyses were conducted to calculate the area under the curve (AUC) for discrimination of the CN control Aβ pathology–negative group from the Aβ pathology–positive MCI (MCI Aβ+) or Alzheimer’s disease group, and DeLong’s test was used to compare the AUCs of different fluid biomarkers. Cohen’s *d* was calculated to estimate the effect size of group differences. Associations between CSF 14-3-3β and longitudinal changes in cognition performance and neuroimaging were tested using linear mixed effects (LME) models. The LME models included an interaction between CSF 14-3-3β and time as the independent variable, and random intercept and random slope with the covariance of the random effects were unstructured. To directly compare effects, all outcome variables in LME models were standardized to *Z*-scores. All LME models included adjustments for age, sex, education years and APOE ε4 status, and adjustments for intracranial volume were also included in models involving imaging of the brain hippocampus. We utilized Cox proportional hazard regression to assess associations between CSF 14-3-3β and risk of progression to Alzheimer’s disease dementia. Initially, univariate analyses were used for the independent variables CSF 14-3-3β, age, sex, education years and APOE ε4 status, and then independent variables with *P* < 0.1 were further entered into a multivariate Cox proportional hazard model to analyse risk of progression to Alzheimer’s disease dementia. Schoenfeld residuals were used to evaluate the proportional hazards assumption. All figures except boxplots were created using Stata version 16 (College Station, TX) statistical software, and boxplots were created using R (version 4.2.0). All statistical analyses were conducted using Stata version 16 (College Station, TX) statistical software, and two-tailed *P* < 0.05 was deemed statistically significant.

## Results

A total of 719 participants with available CSF 14-3-3β measurements from targeted proteomics assays were involved in this study. Participants’ demographic characteristics are summarized in Table 1. Aβ-positive individuals were older and included a higher proportion of APOE ε4 carriers. CSF 14-3-3β peptide 44-51 and peptide 63-70 levels were significantly higher in the Aβ-positive group than the Aβ-negative group. As expected, FDG-PET composite values, brain MRI hippocampus volumes and MMSE scores were significantly lower in the Aβ-positive group. Among the participants in the Aβ-positive group, there were 176 (54.83%) males. In the Aβ-negative group, there were 199 (50%) male participants (Table 1).

**Table 1:** Demographic data of the study population

Variable	Aβ− (*n* = 398)	Aβ+ (*n* = 321)	*P*-value
Age at baseline, years	70.70 (66.20–75.80)	74.30 (69.00–78.80)	<0.001
Male sex, *n* (%)	199 (50.00%)	176 (54.83%)	0.20
Education level, years	16.0 (14.0–18.0)	16.0 (14.0–18.0)	0.016
APOE ε4 status, *n* (%)			<0.001
APOE ε4^−/−^, *n* (%)	297 (74.62%)	98 (30.53%)	
APOE ε4^+/−^, *n* (%)	94 (23.62%)	158 (49.22%)	
APOE ε4^+/+^, *n* (%)	7 (1.76%)	65 (20.25%)	
CSF biomarkers			
14-3-3β,44–51 total_area_ratio	0.014 (0.012–0.018)	0.020 (0.016–0.024)	<0.001
14-3-3β,63–70 total_area_ratio	0.007 (0.006–0.008)	0.009 (0.008–0.011)	<0.001
Brain neuroimaging^a^			
Hippocampus, mm^3^	7508.0 (6827.0–8013.0)	6622.5 (5821.5–7337.0)	<0.001
AV45 PET	1.03 (0.99–1.10)	1.42 (1.30–1.54)	<0.001
FDG-PET composite	1.28 (1.20–1.37)	1.15 (1.04–1.27)	<0.001
Cognitive score			
MMSE	29.0 (28.0–30.0)	27.0 (24.0–29.0)	<0.001
CDR-SB	0.5 (0.0–1.0)	2.0 (1.0–3.5)	<0.001

Data are presented as median (interquartile range) for continuous variables and *n* (%) for categorical variables. CDR-SB, Clinical Dementia Rating Scale-Sum of Boxes; FDG, fluorodeoxyglucose; MMSE, Mini-Mental State Examination.

^a^Reported MRI structural measurements are unadjusted for total intracranial volume.

**Figure 1:**
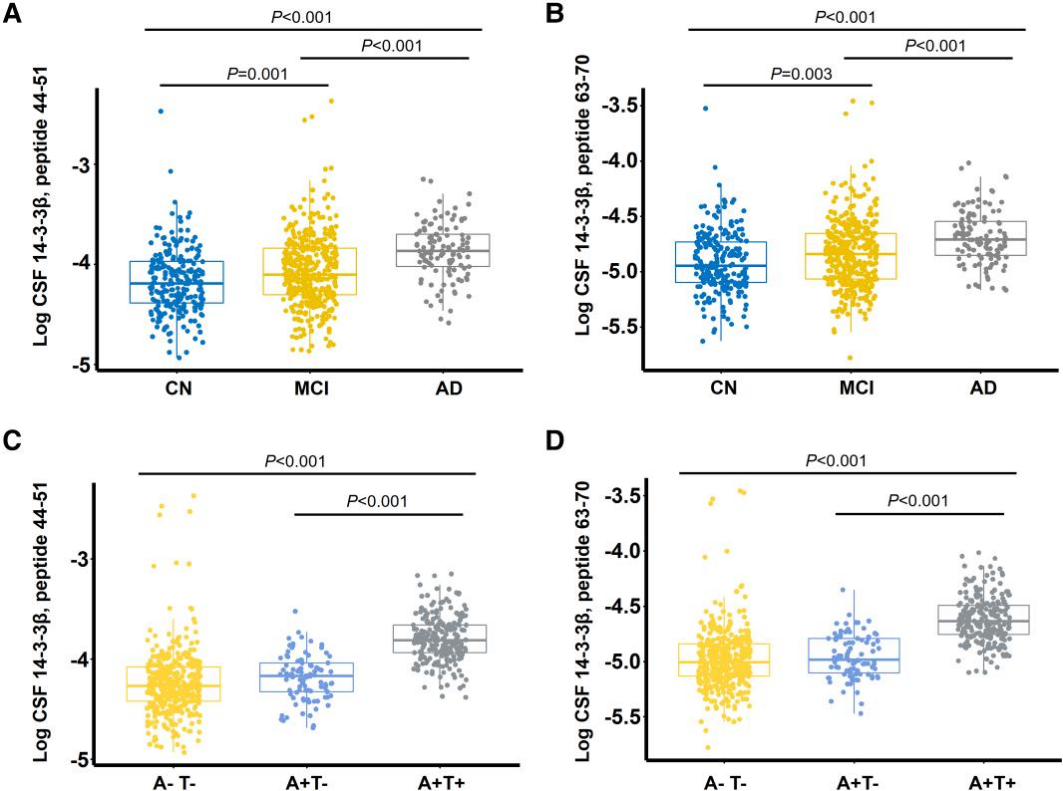
**CSF 14-3-3β according to cognitive states and AT profile.** (**A**, **B**) Comparison of CSF 14-3-3β levels among CN individuals, those with MCI and patients with Alzheimer’s disease dementia. (**C**, **D**) Distribution of CSF 14-3-3β levels in relation to the AT profile. The figures depict the measured levels of two peptides of CSF 14-3-3β, peptide 44-51 (NLLSVAYK; **A**, **C**) and peptide 63-70 (VISSIEQK; **B**, **D**). ‘A’ denotes amyloid pathology, while ‘T’ denotes tau pathology. A/T status positivity was determined using the following cut-off values: CSF p-tau/Aβ42 > 0.028 and CSF p-tau > 27 pg/mL, respectively. One-way ANOVA was used for group comparisons, and significant differences between groups were further explored using the Tukey *post hoc* test. p-tau, phosphorylated tau 181.

The CSF levels of 14-3-3β peptides were higher in the MCI group than in the CN control group (*P* = 0.001), with the highest concentration in the Alzheimer’s disease dementia group (*P* < 0.001; Fig. 1A and B). In this study, we also compared CSF 14-3-3β expression levels between male and female subjects across the three clinical diagnosis groups, and we found no significant differences between the sexes within these groups (Supplementary Fig. 1A). The levels of CSF 14-3-3β peptides were further compared according to participant CSF A/T profiles, and there were no statistically significant differences between the A − T− and A + T− groups. However, the highest CSF 14-3-3β peptide levels were in the A + T+ group, which were significantly higher than the other two groups (*P* < 0.001; Fig. 1C and D).

**Figure 2:**
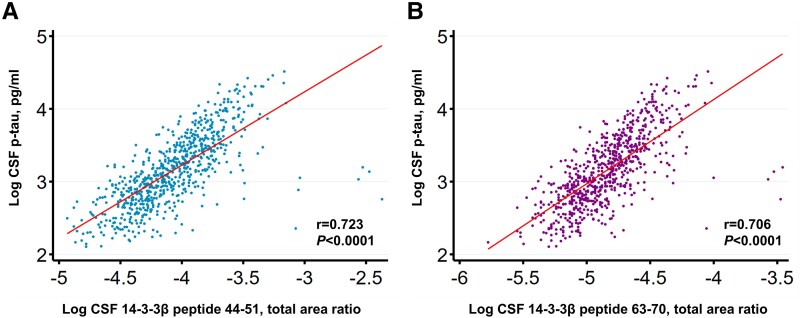
**Correlations of CSF 14-3-3β with CSF biomarker of tau pathology.** Scatter plots illustrating the associations between CSF 14-3-3β and CSF biomarker of tau pathology. (**A**) Log CSF 14-3-3β peptide 44-51 (NLLSVAYK) was positively associated with log CSF p-tau. (**B**) Log CSF 14-3-3β peptide 63-70 (VISSIEQK) was positively associated with log CSF p-tau. The associations between log-transformed fluid biomarkers are depicted using fitted lines, and Pearson correlation was employed to obtain *r*- and *P*-values. p-tau, phosphorylated tau 181.

Our study analysed the associations of CSF 14-3-3β peptides (peptides 44-51 and 63-70) with tau pathology, as measured by CSF p-tau. CSF 14-3-3β peptide 44-51 and peptide 63-70 levels were significantly associated with CSF p-tau (Fig. 2). Associations between CSF 14-3-3β peptide and other biomarkers of Alzheimer’s disease were also studied. CSF 14-3-3β peptide was positively associated with CSF t-tau, synaptic biomarker CSF GAP-43, CSF pTau/Aβ42 ratios and Aβ-PET. There were no significant associations of CSF 14-3-3β peptide with CSF Aβ42, plasma Aβ42/40, plasma p-tau and plasma NfL (Table 2). Using linear regression analyses, we found no significant interactions between sex and amyloid-β pathology (Aβ-PET × sex: *β* = 0.041, *P* = 0.5), tau pathology (CSF p-tau × sex: *β* = 0.004, *P* = 0.095) or MMSE scores (MMSE × sex: *β* = 0.002, *P* = 0.486), as illustrated in Supplementary Fig. 1B–D. These results indicate that sex does not influence the associations between CSF 14-3-3β and the studied biomarkers of Alzheimer’s disease and cognitive status.

**Table 2:** Associations between CSF 14-3-3β and other biomarkers

	Outcome—CSF 14-3-3β, peptide 44-51		
Predictor	*β* coefficient	*P*-value	*P*_adj_-value
CSF Aβ42	−0.016	0.715	0.767
CSF pTau/Aβ42 ratios	0.458	<0.001	<0.001
CSF p-tau	0.573	<0.001	<0.001
CSF t-tau	0.590	<0.001	<0.001
CSF GAP-43	0.413	<0.001	<0.001
Plasma Aβ42/40	−0.020	0.767	0.767
Plasma p-tau	0.067	0.071	0.091
Plasma NfL	0.076	0.058	0.087
PET AV45	0.170	<0.001	<0.001

Linear regression models were employed to investigate the associations between CSF 14-3-3β and other biomarkers, and all biomarkers were standardized to *Z*-scores to facilitate a direct comparison of their effects. Linear regression models were adjusted for age, sex, years of education and APOE ε4 genotype. *P*_ad_ denotes the *P*-values adjusted for multiple comparisons using the Benjamini–Hochberg method. p-tau, phosphorylated tau 181; t-tau, total tau; NfL, neurofilament light.

**Figure 3:**
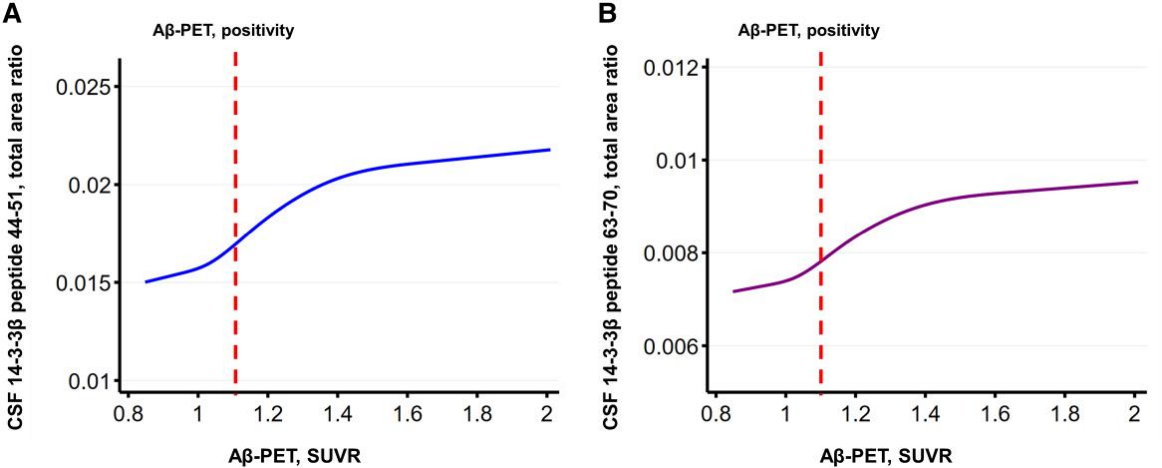
**CSF 14-3-3β trajectories demonstrate early increases with Aβ-PET burden.** Cubic spline models depict the patterns of change in (**A**) CSF 14-3-3β peptide 44-51 (NLLSVAYK) and (**B**) CSF 14-3-3β peptide 63-70 (VISSIEQK) as the global Aβ-PET SUVRs increase, using Aβ-PET SUVR as an indicator of Alzheimer’s disease progression. Specifically, the trajectories represent the estimated CSF 14-3-3β peptide concentrations as a function of the Aβ-PET SUVR. Analysis based on 531 participants. The vertical dashed lines represent the Aβ-PET positivity cut-off. SUVR, standard uptake value ratio.

To assess the changes of CSF 14-3-3β concentrations over the disease course of Alzheimer’s disease, cubic spline models were applied to model the trajectories of CSF 14-3-3β concentrations across the Alzheimer’s disease continuum, and Aβ-PET SUVRs were used as proxies for disease progression. In order to model the disease course over the Alzheimer’s disease continuum, our analyses excluded cognitively impaired participants who were Aβ-PET negative. The trajectories demonstrated that there was an early increase of CSF 14-3-3β peptide 44-51 and peptide 63-70 concentrations prior to development of Aβ-PET–positive status, and that the concentrations continued to rise after reaching the threshold of Aβ-PET positivity, then gradually came to a plateau (Fig. 3A and B).

**Figure 4:**
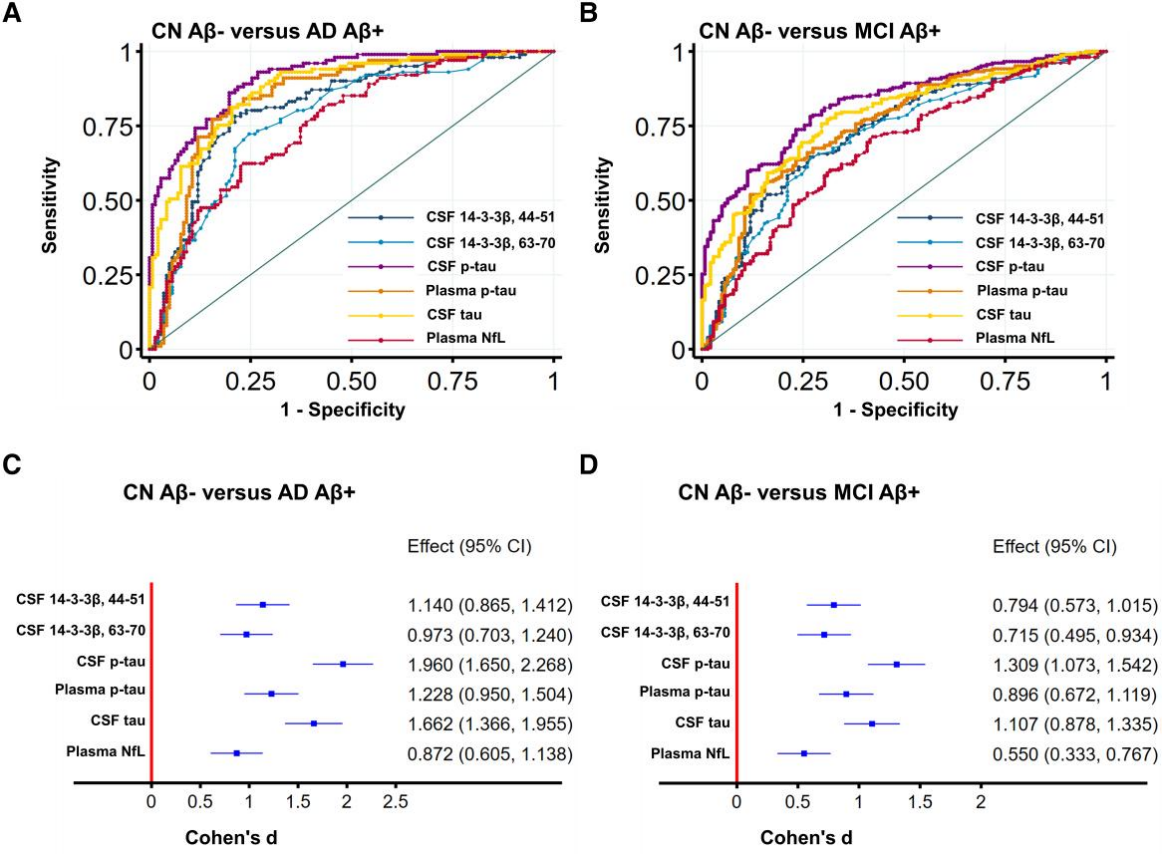
**Comparing the performance of CSF 14-3-3β peptides and other fluid biomarkers.** (**A**) ROC analysis was conducted to evaluate the accuracy of CSF 14-3-3β peptides and other fluid biomarkers in distinguishing the CN Aβ− from the Alzheimer’s disease Aβ+. Aβ status was defined by Aβ-PET. Analysis based on 243 participants. (**B**) ROC analysis was performed to assess the accuracy of CSF 14-3-3β peptides and other fluid biomarkers in distinguishing CN Aβ− from the MCI Aβ+, with Aβ status determined by Aβ-PET. Analysis based on 348 participants. (**C**) The effect sizes of CSF 14-3-3β peptides and other fluid biomarkers between the CN Aβ− and Alzheimer’s disease Aβ+ groups. Cohen’s *d* was calculated to estimate the effect size of differences between groups. (**D**) The effect sizes of CSF 14-3-3β peptides and other fluid biomarkers between the CN Aβ− and MCI Aβ+ groups. To estimate the effect size of group differences, Cohen’s *d* was calculated. AD, Alzheimer’s disease; AUC, area under the curve; CN, cognitively normal; MCI, mild cognitive impairment; NfL, neurofilament light; p-tau, phosphorylated tau 181; ROC, receiver operating characteristic; t-tau, total tau.

We next compared the diagnostic accuracy of CSF 14-3-3β peptides (peptides 44-51 and 63-70) and other fluid biomarkers to differentiate the CN control Aβ pathology–negative group (CN Aβ−) from the Aβ pathology–positive Alzheimer’s disease dementia group (Alzheimer’s disease Aβ+), using Aβ pathology measured by amyloid PET. ROC analyses were conducted, and the AUCs for each fluid biomarker were compared using DeLong’s test. The accuracy of CSF 14-3-3β peptide 44-51 was significantly lower than that of CSF p-tau and CSF t-tau but not significantly different from plasma p-tau and plasma NfL (Fig. 4A; Supplementary Table 1). As for comparison between the CN Aβ− group and the Alzheimer’s disease Aβ+ group, CSF p-tau (Cohen’s *d* = 1.96) and CSF t-tau (Cohen’s *d* = 1.662) showed the highest degree of change, followed by plasma p-tau (Cohen’s *d* = 1.228), CSF 14-3-3β peptide 44-51 (Cohen’s *d* = 1.14), CSF 14-3-3β peptide 63-70 (Cohen’s *d* = 0.973) and plasma NfL (Cohen’s *d* = 0.872; Fig. 4C). In regard to the differentiation of the CN Aβ− from the MCI Aβ+ group, the diagnostic accuracy of CSF 14-3-3β peptide 44-51 was significantly lower than that of CSF p-tau and CSF t-tau but not significantly different from that of plasma p-tau, CSF 14-3-3β peptide 63-70 or plasma NfL (Fig. 4B; Supplementary Table 1). Comparison between the CN Aβ− group and MCI Aβ+ group demonstrated the highest degrees of change in CSF p-tau (Cohen’s *d* = 1.309) and CSF t-tau (Cohen’s *d* = 1.107), followed by plasma p-tau (Cohen’s *d* = 0.896), CSF 14-3-3β peptide 44-51 (Cohen’s *d* = 0.794) and CSF 14-3-3β peptide 63-70 (Cohen’s *d* = 0.715), while plasma NfL (Cohen’s d = 0.55) showed the lowest degree of change (Fig. 4D).

**Figure 5:**
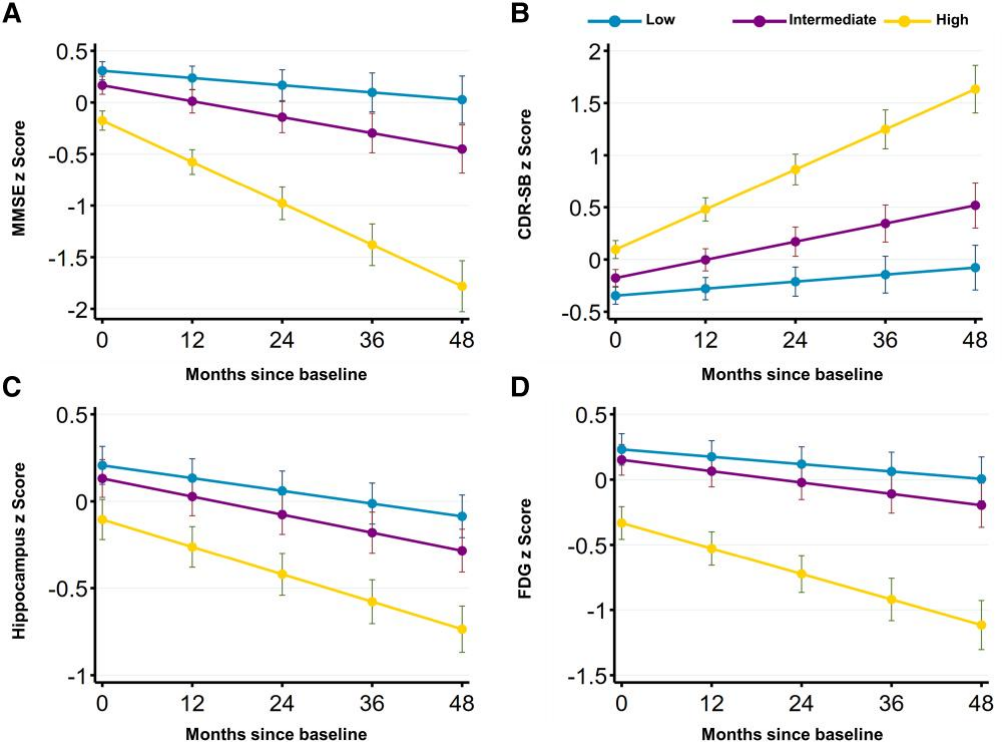
**CSF 14-3-3β levels associated with longitudinal cognitive and neuroimaging changes.** LME models were used to compare MMSE scores (**A**), CDR-SB scores (**B**), hippocampus volumes (**C**), and FDG-PET composite ROI changes (**D**) between CSF 14-3-3β groups over time. CSF 14-3-3β levels were categorized into three tertiles: low-level (243 participants), intermediate-level (242 participants) and high-level (234 participants) groups. Supplementary Tables 2–5 provide information on the baseline levels (intercept) and temporal alterations (slope) of the respective trajectories. All outcome variables were standardized to *Z*-scores to facilitate comparisons. CDR-SB, Clinical Dementia Rating Scale-Sum of Boxes; FDG, fluorodeoxyglucose; MMSE, Mini-Mental State Examination; ROI, region of interest.

Next, we investigated whether baseline CSF 14-3-3β peptide levels were associated with cognitive changes, in terms of MMSE scores and CDR-SB scores, over 4 years of follow-up. According to the baseline distribution of CSF 14-3-3β peptide 44-51 levels, participants were subdivided into low-, intermediate- and high-level groups. Intermediate and high levels of CSF 14-3-3β peptide were associated with accelerated reductions in MMSE scores and increases in CDR-SB scores over time (Fig. 5A and B; Supplementary Tables 2 and 3). We also evaluated whether baseline CSF 14-3-3β peptide levels were associated with longitudinal neuroimaging finding changes, which included hippocampus volumes and FDG-PET values. Intermediate and high levels of CSF 14-3-3β were associated with faster reductions of hippocampus volumes during follow-up, and high levels of CSF 14-3-3β were associated with a greater decline of FDG-PET values over time (Fig. 5C and D; Supplementary Tables 4 and 5).

**Figure 6:**
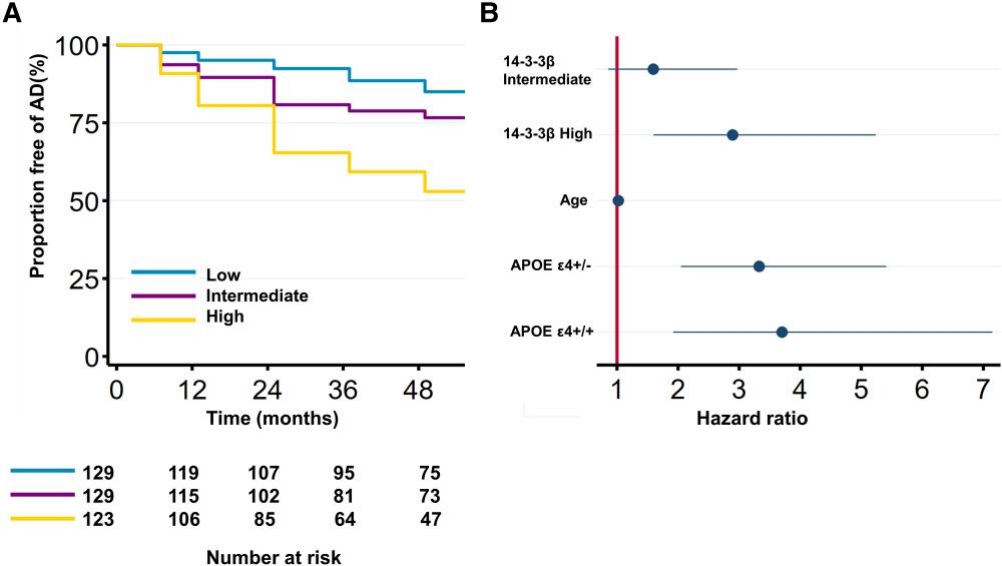
**CSF 14-3-3β levels and progression from MCI to Alzheimer's disease dementia.** (**A**) The Kaplan–Meier curves illustrate the progression from MCI to Alzheimer’s disease dementia for the low, intermediate and high CSF 14-3-3β tertiles. (**B**) A multivariate Cox regression analysis was performed to assess the progression from MCI to Alzheimer’s disease dementia. The model included the CSF 14-3-3β tertiles, age and APOE ε4 genotype. The associations are represented as HR along with their respective 95% confidence intervals. Analysis based on 381 participants. MCI, mild cognitive impairment.

Kaplan–Meier curves showed the probability of progression to Alzheimer’s disease dementia in three MCI patient groups subdivided into tertiles of the CSF 14-3-3β peptide 44-51 level distribution, and the probability of development of Alzheimer’s disease dementia in MCI patients was statistically different among the three groups (log-rank *P* < 0.0001; Fig. 6A). In the multivariate Cox proportional hazards model adjusted by age and APOE ε4 status, compared to the low-level CSF 14-3-3β group, there was a significantly increased risk of progression to Alzheimer’s disease dementia in MCI patients in the high-level CSF 14-3-3β group at baseline [hazard ratio (HR) = 2.894 (1.599–5.238), *P* < 0.001; Fig. 6B; Supplementary Table 6].

## Discussion

The 14-3-3 proteins are a family of regulatory proteins that play critical roles in various cellular processes. In previous studies, the CSF 14-3-3 isoform β was identified as one of the specific neuronal biomarkers for the diagnosis of sporadic Creutzfeldt–Jakob disease, and assay of CSF 14-3-3β protein is used routinely to aid in the clinical diagnosis of sporadic Creutzfeldt–Jakob disease worldwide.^8–11^ Alzheimer’s disease is a neurodegenerative disorder characterized pathologically by β-amyloid plaques and neurofibrillary tangles containing tau, and several studies have found that 14-3-3 proteins are present in the neurofibrillary tangles of Alzheimer’s disease brains.^26,27^ In this study, CSF 14-3-3β levels were measured by targeted proteomics assay. Targeted proteomics assay is a specialized analytical technique that employs MS to detect and quantify specific proteins of interest in biological samples, such as blood, urine or CSF. Compared to traditional protein quantification methods such as western blot or ELISA, targeted proteomics assay does not require antibodies. Unlike integrative proteomics, which can measure thousands of proteins without any prior knowledge, targeted proteomics assay is a hypothesis-driven technique requiring prior knowledge of these proteins of interest.^28,29^ A previous study using integrative proteomics identified hundreds of altered proteins in the CSF samples of Alzheimer’s disease patients, including increased CSF 14-3-3β levels.^13^ The present study involved 719 participants with available baseline CSF 14-3-3β measurements and explored the relationship between CSF 14-3-3β levels and various participant groups, including those with MCI, Alzheimer’s disease dementia and CN controls. This study also examined the relationship between CSF 14-3-3β levels and the presence of amyloid-beta and tau pathologies. Our findings showed that CSF 14-3-3β levels were significantly higher in the MCI group compared to the CN control group, and the highest CSF 14-3-3β levels were observed in the Alzheimer’s disease dementia group. These findings suggest that increased CSF 14-3-3β levels may be associated with cognitive decline and could potentially be a marker for monitoring disease progression.

To further investigate this relationship, we compared the CSF 14-3-3β levels across different A/T profile groups (A − T−, A + T− and A + T+). No significant differences were found between the AT− and A + T− groups, and the highest levels of CSF 14-3-3β were observed in the A + T + group, which had significantly higher CSF 14-3-3β levels compared to the other two groups. This result suggests an association between elevated CSF 14-3-3β levels and the presence of both amyloid-beta and tau pathologies, which are indicative of an advanced pathological stage of Alzheimer’s disease.^30^ Consequently, results of this study imply that CSF levels of 14-3-3β may serve as a potential biomarker for the presence and severity of the core pathologies of Alzheimer’s disease.

In this study, we demonstrated that CSF 14-3-3β levels are associated with CSF p-tau levels. In Alzheimer’s disease, neurofibrillary tangles are formed by the abnormal aggregation of hyperphosphorylated tau protein, and previous studies have shown that 14-3-3 proteins are present in the neurofibrillary tangles of Alzheimer’s disease patients.^26,27^ In brain extracts, tau interacts with 14-3-3 isoforms beta and zeta.^31^ Since CSF p-tau is a biomarker of tau pathology, the correlation between CSF 14-3-3β and CSF p-tau indicates that CSF 14-3-3β is also associated with tau pathology. Previous studies have found that the synaptic biomarker CSF GAP-43 is associated with tau pathology in Alzheimer’s disease,^18,32^ and in this study, we also observed that CSF 14-3-3β is associated with CSF GAP-43, highlighting the combination of tau pathology, synaptic dysfunction and 14-3-3β pathological changes of Alzheimer’s disease. This study found that CSF 14-3-3β levels were significantly higher in the Aβ-positive group compared to the Aβ-negative group. Moreover, CSF 14-3-3β levels showed a positive correlation with Aβ-PET SUVR. Our study also demonstrated an association between CSF 14-3-3β and CSF pTau/Aβ42 ratios, and a previous study highlighted the superiority of CSF pTau/Aβ42 ratios over CSF Aβ42 alone in aligning with Aβ-PET results.^16^ Collectively, these findings indicate that CSF 14-3-3β levels are also related to brain amyloidosis.

This study assessed the changes in CSF 14-3-3β concentrations over the disease course of Alzheimer’s disease by examining their trajectories across the Alzheimer’s disease continuum. We used Aβ-PET SUVR as a proxy for disease progression,^33^ and to ensure an accurate representation of the Alzheimer’s disease continuum, Aβ-PET–negative cognitively impaired participants were excluded from the analyses. The trajectories of CSF 14-3-3β peptide 44-51 and peptide 63-70 concentrations showed an early increase prior to reaching Aβ-PET–positive status. This phenomenon suggests that the levels of CSF 14-3-3β may start to rise even before the brain has significant Aβ plaque accumulation. After crossing the threshold of Aβ-PET positivity, CSF 14-3-3β levels continued to rise, indicating that their levels increase as the disease progresses. However, CSF 14-3-3β levels eventually reached a plateau, meaning that the increase in CSF 14-3-3β slows down or stabilizes at a certain time point in the disease course. These findings are important for understanding the role of 14-3-3β in the pathophysiology of Alzheimer’s disease. The fact that its concentrations increase before Aβ-PET positivity is reached and continue to rise as the disease progresses might suggest that CSF 14-3-3β could potentially serve as an early biomarker for Alzheimer’s disease or that 14-3-3β may be involved in the underlying disease mechanisms, and further study is needed to confirm and understand these observations.

The present study compared the diagnostic accuracy of several fluid biomarkers in distinguishing CN Aβ− individuals from Alzheimer’s disease Aβ+ or MCI Aβ+ patients, using amyloid PET to define Aβ status. The biomarkers compared included CSF 14-3-3β peptides (peptides 44-51 and 63-70), CSF p-tau and CSF t-tau, plasma p-tau and plasma NfL. Our results suggest that while CSF 14-3-3β is less accurate than CSF p-tau and CSF t-tau in discriminating CN Aβ− from Alzheimer’s disease Aβ+ or MCI Aβ+ individuals, CSF 14-3-3β performs similarly to plasma p-tau and plasma NfL. These findings suggest that while CSF 14-3-3β may not be as accurate as CSF p-tau and CSF t-tau in distinguishing CN Aβ− from Alzheimer’s disease Aβ+ or MCI Aβ+ groups, it may still be a promising biomarker for Alzheimer’s disease diagnosis, particularly when compared to plasma biomarkers such as plasma p-tau and plasma NfL. In this study, the comparison of diagnostic accuracy of CSF 14-3-3β with multiple fluid biomarkers also provides a comprehensive illustration of CSF 14-3-3β’s diagnostic performance in Alzheimer’s disease.

Our study also investigated whether baseline levels of CSF 14-3-3β could predict changes in cognitive function, as well as changes on neuroimaging over time. The results showed that, compared to those with low levels of CSF 14-3-3β, individuals with intermediate and high levels of CSF 14-3-3β had accelerated reductions in MMSE scores and increases in CDR-SB scores, indicating that higher baseline levels of CSF 14-3-3β are associated with faster cognitive decline over time. Additionally, these individuals also had faster reductions in hippocampus volumes and greater declines in FDG-PET values, suggesting that higher baseline levels of CSF 14-3-3β are associated with greater neurodegeneration and neuronal dysfunction over time. These findings thus indicate that higher baseline levels of CSF 14-3-3β may predict accelerated cognitive decline and more pronounced neurodegeneration in individuals at risk for progression to dementia. However, further studies are needed to explore underlying mechanisms for the observed associations between baseline CSF 14-3-3β levels and longitudinal changes in cognitive function and on neuroimaging.

MCI is a condition characterized by cognitive impairment that is greater than expected for an individual’s age and education level but not severe enough to meet the criteria for dementia. However, many individuals with MCI will eventually progress to Alzheimer’s disease dementia.^34^ Identifying individuals with MCI who are likely to progress to Alzheimer’s disease dementia could help guide more targeted interventions and follow-up strategies. Our study showed that the probability of conversion to Alzheimer’s disease dementia was significantly different among the three MCI patient groups based on their CSF 14-3-3β distributions, with high levels of CSF 14-3-3β associated with increased risk of progression to Alzheimer’s disease dementia. With a previous study demonstrating that CSF Aβ42, t-tau and p-tau levels are associated with the risk of progression to Alzheimer’s disease dementia in MCI patients,^35^ our findings suggest that measuring CSF 14-3-3β levels in MCI patients might also be a useful tool for identifying those at the highest risk of developing Alzheimer’s disease dementia.

The findings of this study are consistent with previous research that demonstrated the potential of CSF 14-3-3β as a biomarker for neurodegenerative diseases, such as Creutzfeldt–Jakob disease.^8,9^ Our study provides evidence that CSF 14-3-3β levels are increased in Alzheimer’s disease and are associated with other biomarkers of the disease, such as biomarkers of tau pathology and the synaptic biomarker CSF GAP-43.^18,20,36^ The association between CSF 14-3-3β levels and tau pathology is particularly noteworthy, as it highlights the potential of 14-3-3β as a biomarker of tau-related neurodegeneration. Additionally, our study showed an early increase in CSF 14-3-3β concentrations before Aβ-PET–positive status was reached, suggesting that CSF 14-3-3β may be a useful biomarker for preclinical Alzheimer’s disease patients.

The present study has several limitations. First, due to lack of data from patients with other neurodegenerative disorders, such as dementia with Lewy bodies, frontotemporal dementia, progressive supranuclear palsy and corticobasal syndrome, the current study is limited in its ability to assess the specificity of CSF 14-3-3β for Alzheimer’s disease. Second, the CSF biomarkers were employed as proxies of Alzheimer’s disease pathology, and it should be noted that autopsy remains the gold standard for assessing Alzheimer’s disease pathology. Third, in this study, CSF 14-3-3β levels were measured by targeted proteomics assay using mass spectrometers. However, MS is not commonly used in clinical practice for analysis of CSF biomarker levels, and other traditional protein quantification methods such as ELISA assay of CSF 14-3-3β should be explored to delineate the characteristics of CSF 14-3-3β in Alzheimer’s disease patients. Fourth, we utilized CSF pTau181 and plasma pTau181 as markers for tau pathology. However, other phosphorylation sites, such as pTau217 (data not available in ADNI), could be more effective biomarkers.^37^

In conclusion, alongside how CSF 14-3-3β is routinely used clinically as a biomarker to aid in the diagnosis of sporadic Creutzfeldt–Jakob disease, our study provides additional evidence that CSF 14-3-3β levels are increased in Alzheimer’s disease and that they are associated with other biomarkers of the disease, such as biomarkers of tau pathology and synaptic biomarkers. Though the diagnostic accuracy of CSF 14-3-3β was moderate compared to CSF p-tau and CSF t-tau in distinguishing CN Aβ− from Alzheimer’s disease Aβ+ individuals, baseline CSF 14-3-3β levels were associated with progressive decline of cognitive function over time. These findings suggest that CSF 14-3-3β may be a useful clinical biomarker for Alzheimer’s disease and could potentially aid in early diagnosis and prediction of disease progression.

## Supplementary material

Supplementary material is available at *Brain Communications* online.

## Supplementary Material

fcad312_Supplementary_Data

## Data Availability

Data used in this study were originally obtained from the online repository of Alzheimer’s Disease Neuroimaging Initiative (ADNI; http://adni.loni.usc.edu/).
