# Supplementary material for: CSF 14-3-3β is associated with progressive cognitive decline in Alzheimer’s disease
Source: Brain Commun. 2023 Nov 22;5(6):fcad312. doi: 10.1093/braincomms/fcad312 (PMC10684297; doi:10.1093/braincomms/fcad312)
Supplement: fcad312_Supplementary_Data [file fcad312_supplementary_data.docx]

**Supplementary Table 1. ROC analysis to differentiate CN** **Aβ- from AD** **Aβ+ and MCI** **Aβ+ individuals (****Aβ status was defined by amyloid PET)**

|  | **CN** **Aβ- versus AD** **Aβ+** | | | **CN** **Aβ- versus MCI** **Aβ+** | | |
| --- | --- | --- | --- | --- | --- | --- |
| **Biomarker** | **AUC** | ***P*-value** | ***P*_adj_ value** | **AUC** | ***P*-value** | ***P*_adj_ value** |
| CSF 14-3-3β, 44-51 | 0.819 |  |  | 0.733 |  |  |
| versus  CSF 14-3-3β, 63-70 | 0.776 | **0.002** | 0.003 | 0.713 | 0.070 | 0.117 |
| versus CSF p-tau | 0.914 | **<0.001** | 0<0.001 | 0.826 | **<0.00**1 | <0.001 |
| versus CSF t-tau | 0.879 | **0.002** | 0.003 | 0.783 | **0.013** | 0.033 |
| versus plasma p-tau | 0.849 | 0.417 | 0.417 | 0.754 | 0.573 | 0.573 |
| versus plasma NfL | 0.756 | 0.102 | 0.128 | 0.673 | 0.103 | 0.129 |

The DeLong test was performed to compare the AUC values of CSF 14-3-3β peptide 44-51 and other fluid biomarkers. Positive Aβ status was defined as amyloid PET SUVR**>** 1.11. *P*_adj_ indicates *P*-values that were corrected for multiple comparisons with the Benjamini-Hochberg method. *P*-values less than 0.05 were deemed statistically significant and are emphasized in bold. Abbreviations: ROC, receiver operating characteristic; AUC, area under the curve; CN Aβ-, cognitively normal Aβ pathology negative group; AD Aβ+, Aβ pathology positive Alzheimer’s disease dementia group; MCI Aβ+, Aβ pathology positive mild cognitive impairment group; CSF, cerebrospinal fluid; p-tau, phosphorylated tau 181; t-tau, total tau; NfL, neurofilament light; PET, positron emission tomography.

**Supplementary Table 2. Associations of MMSE Z scores with CSF 14-3-3β levels**

|  | **Low** | **Intermediate** | **High** |
| --- | --- | --- | --- |
| Intercept, β | 0.307 | 0.166 | -0.175 |
| Intercept, *P*-value | NA | **0.026** | **<0.001** |
| Slope, β | -0.002 | -0.006 | -0.013 |
| Slope, *P*-value | NA | **0.009** | **<0.001** |

Associations of baseline (intercept) and longitudinal (slope) MMSE Z scores with CSF 14-3-3β levels were tested using linear mixed effects models, adjusted for age, sex, education years, and APOE ε4 genotype. CSF 14-3-3β levels were categorized into low, intermediate, and high tertiles, using the low-level group as the reference group for comparisons. *P*-values less than 0.05 were deemed statistically significant and are emphasized in bold. Abbreviations: MMSE, Mini-Mental State Examination.

**Supplementary Table 3. Associations of CDR-SB Z scores with CSF 14-3-3β levels**

|  | **Low** | **Intermediate** | **High** |
| --- | --- | --- | --- |
| Intercept, β | -0.346 | -0.177 | 0.095 |
| Intercept, *P*-value | NA | **0.004** | **<0.001** |
| Slope, β | 0.006 | 0.014 | 0.032 |
| Slope, *P*-value | NA | **<0.001** | **<0.001** |

Associations of baseline (intercept) and longitudinal (slope) CDR-SB Z scores with CSF 14-3-3β levels were tested using linear mixed effects models, adjusted for age, sex, education years, and APOE ε4 genotype. CSF 14-3-3β levels were categorized into low, intermediate, and high tertiles, with the low-level group used as the reference group for comparisons. *P*-values less than 0.05 were deemed statistically significant and are emphasized in bold. Abbreviations: CDR-SB, Clinical Dementia Rating Scale-Sum of Boxes.

**Supplementary Table 4. Associations of FDG-PET Z scores with CSF 14-3-3β levels**

|  | **Low** | **Intermediate** | **High** |
| --- | --- | --- | --- |
| Intercept, β | 0.231 | 0.151 | -0.333 |
| Intercept, *P*-value | NA | **0.355** | **<0.001** |
| Slope, β | -0.005 | -0.007 | -0.016 |
| Slope, *P*-value | NA | **0.180** | **<0.001** |

Associations of baseline (intercept) and longitudinal (slope) FDG-PET composite ROIs Z scores with CSF 14-3-3β levels were tested using linear mixed effects models, adjusted for age, sex, education years, and APOE ε4 genotype. CSF 14-3-3β levels were categorized into low, intermediate, and high tertiles, with the low-level group used as the reference group for comparisons. *P*-values less than 0.05 were deemed statistically significant and are emphasized in bold. Abbreviations: FDG, 18F-fluorodeoxyglucose; ROI, region of interest.

**Supplementary Table 5. Associations of hippocampus volume Z scores with CSF 14-3-3β levels**

|  | **Low** | **Intermediate** | **High** |
| --- | --- | --- | --- |
| Intercept, β | 0.207 | 0.131 | -0.104 |
| Intercept, *P*-value | NA | **0.341** | **<0.001** |
| Slope, β | -0.006 | -0.009 | -0.013 |
| Slope, *P*-value | NA | **<0.001** | **<0.001** |

Associations of baseline (intercept) and longitudinal (slope) hippocampus volume Z scores with CSF 14-3-3β levels were tested using linear mixed effects models, adjusted for age, sex, education years, APOE ε4 genotype, and intracranial volume. CSF 14-3-3β levels were categorized into low, intermediate, and high tertiles, with the low-level group used as the reference group for comparisons. *P*-values less than 0.05 were deemed statistically significant and are emphasized in bold.

**Supplementary Table 6.** **Univariate and** **multivariate Cox regression analysis of progression from MCI to AD dementia.**

**a**, HR (95% CI) calculated using univariate Cox regression analyses. **b**, HR (95% CI) calculated using multivariate Cox regression analyses, adjusted for age and APOE ε4 genotype. Abbreviations: AD, Alzheimer’s disease; CI, confidence interval; HR, hazard ratio; MCI, mild cognitive impairment.

|  | **Crude** |  | **Adjusted** |  |
| --- | --- | --- | --- | --- |
| **Variable** | **HR (95% CI)^a^** | ***P*-value** | **HR (95% CI)^b^** | ***P*-value** |
| CSF 14-3-3β |  |  |  |  |
| Low | Reference | - | Reference | - |
| Intermediate | 1.895 (1.029 - 3.489) | 0.040 | 1.595 (0.856-2.969) | 0.141 |
| High | 3.882 (2.208 - 6.823) | <0.001 | 2.894 (1.599-5.238) | <0.001 |
| Age at baseline | 1.023 (0.996 - 1.051) | 0.090 | 1.022 (0.992-1.053) | 0.158 |
| Education years | 0.979 (0.907 - 1.058) | 0.597 | - | - |
| APOE ε4 status |  |  |  |  |
| APOE ε4-/- | Reference | - | Reference | - |
| APOE ε4+/- | 3.454 (2.148 - 5.554) | <0.001 | 3.328 (2.048-5.408) | <0.001 |
| APOE ε4+/+ | 3.815 (2.035 - 7.153) | <0.001 | 3.704 (1.920-7.146) | <0.001 |
| Sex |  |  |  |  |
| Female | Reference | - | - | - |
| Male | 1.190 (0.791 - 1.792) | 0.403 | - | - |

**Supplementary Figure 1. Sex does not influence CSF 14-3-3β’s association with cognitive status and biomarkers of AD.**

**
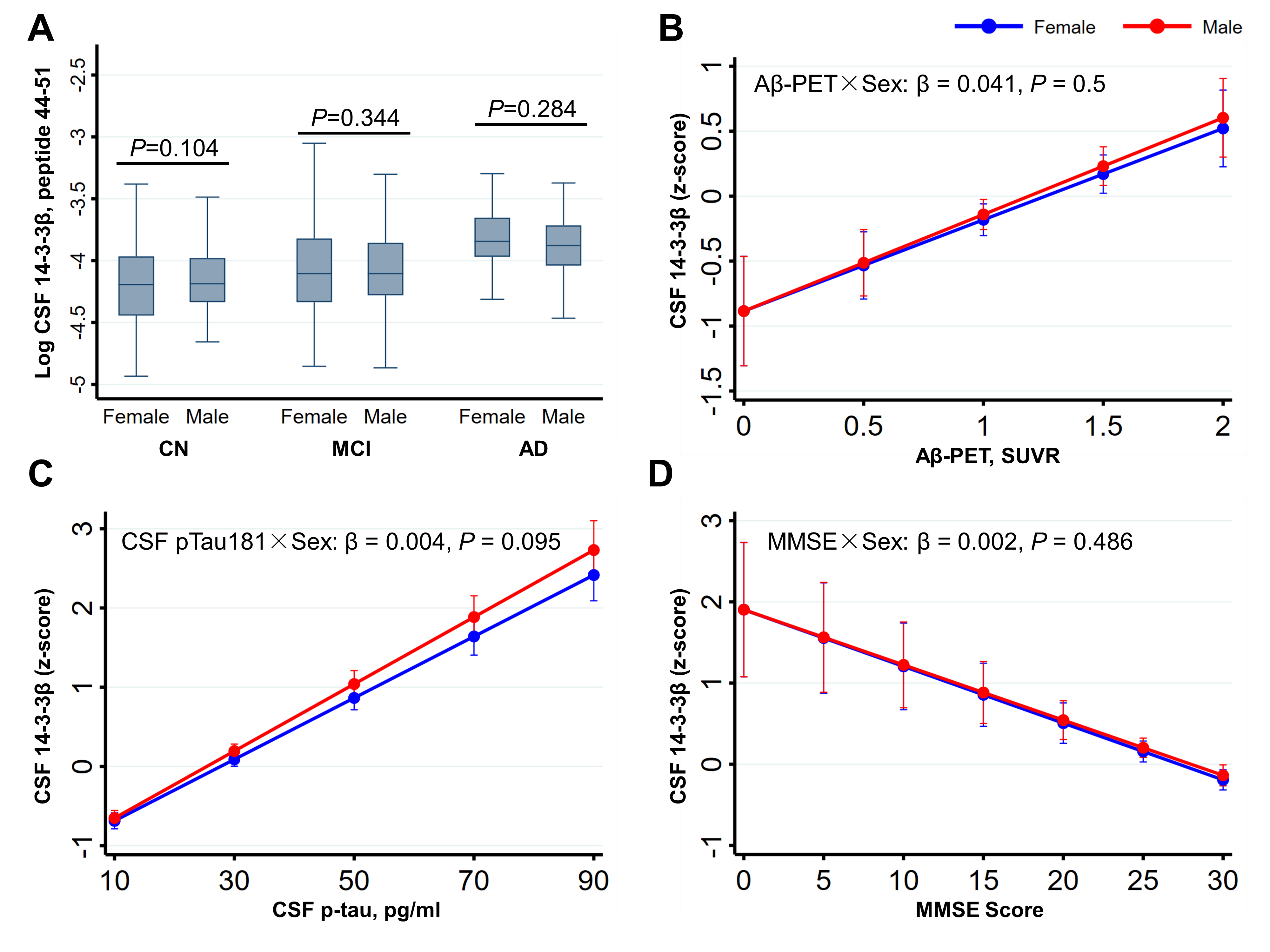
**

(**A**), Comparison of CSF 14-3-3β levels between males and females in the cognitively normal (CN) group, the mild cognitive impairment (MCI) group, and the Alzheimer’s disease (AD) dementia group. (**B**), Linear regression analyses (adjusted for age, education years and APOE ε4 status) revealed that there was no interaction between amyloid-β pathology and sex on CSF 14-3-3β levels. (**C**), Linear regression analyses (adjusted for age, education years and APOE ε4 status) showed no significant interaction between CSF p-tau and sex on CSF 14-3-3β levels. (**D**), Linear regression analyses (adjusted for age, education years and APOE ε4 status) demonstrated no significant interaction between MMSE score and sex on CSF 14-3-3β levels. Abbreviations: AD, Alzheimer’s disease; MMSE, Mini-Mental State Examination; p-tau, phosphorylated tau 181.
